# Saikosaponin A-Induced Gut Microbiota Changes Attenuate Severe Acute Pancreatitis through the Activation of Keap1/Nrf2-ARE Antioxidant Signaling

**DOI:** 10.1155/2020/9217219

**Published:** 2020-11-01

**Authors:** Jing Li, Jinfeng Han, Juan Lv, Shiji Wang, Lai Qu, Yanfang Jiang

**Affiliations:** ^1^Department of Intensive Care Unit, The First Hospital of Jilin University, Changchun 130021, China; ^2^Genetic Diagnosis Center, The First Hospital of Jilin University, Changchun 130021, China

## Abstract

**Objective:**

Severe acute pancreatitis (SAP) is a serious and life-threatening disease associated with multiple organ failure and a high mortality rate and is accompanied by distinct oxidative stress and inflammatory responses. Saikosaponin A has strong antioxidant properties and can affect the composition of gut microbiota. We sought to determine the effects of Saikosaponin A interventions on SAP by investigating the changes of gut microbiota and related antioxidant signaling.

**Methods:**

A SAP model was established in Sprague-Dawley (SD) rats through the injection of sodium taurocholate into the biliopancreatic duct and confirmed by elevated levels of serum lipase and amylase. The model was fed a standard diet either with saline solution or with Saikosaponin A. Fecal microbiota transplantation (FMT) from Saikosaponin A-induced rats into the rat model was performed to test the effects of gut microbiota. The composition of gut microbiota was analyzed by using 16S rRNA gene sequencing. We measured apoptotic status, inflammatory biomarkers, and Keap1-Nrf2-ARE ((Kelch-like ECH-associated protein 1) nuclear factor erythroid 2-related factor 2-antioxidant response element) antioxidant signaling.

**Results:**

Saikosaponin A intervention attenuated SAP lesions and reduced the levels of serum amylase and lipase, oxidative stress, and inflammatory responses by reducing pathological scores and affecting the serum level of oxidative and inflammatory factors. Meanwhile, the expression of Keap1-Nrf2-ARE was increased. Saikosaponin A intervention improved microbiota composition by increasing the relative abundance of Lactobacillus and Prevotella species. FMT resulted in similar results as those caused by the Saikosaponin A intervention, suggesting Saikosaponin A may exert its function via the improvement of gut microbiota composition.

**Conclusions:**

Saikosaponin A-induced gut microbiota changes attenuate SAP progression in the rat model and may be a potential natural drug for adjuvant treatment of SAP. Further work is needed to clear up the points.

## 1. Introduction

Pancreatitis is a leading complication of gastrointestinal diseases, and often initiates and exacerbates systemic inflammatory responses. The mortality rate of pancreatitis is between 1.5% and 4.2% according to the previous report [1]. Pancreatitis development will lead to the release of inflammatory indicators and cytokines, which lead to intestinal barrier damage [2]. Pancreatitis increases intestinal permeability and facilitates bacterial infection, resulting in the damage of intestinal barrier [3]. Pancreatitis is often involved with lung injury [4, 5], liver disease [6], and other organ failure [7, 8].

Natural products have been found to be effective in the prevention of pancreatitis risk [9, 10]. *Radix bupleuri* is a common Chinese herb and shows anti-inflammatory properties in the prevention of SAP progression [11]. Saikosaponin A is one of the most effective components of *Radix bupleuri* roots [12, 13]. The previous work indicated that Saikosaponin A attenuated hyperlipidemic pancreatitis in an animal model by improving lipid metabolism and preventing the release of proinflammatory cytokines via the NF-kappaB signaling [14]. However, its function on SAP remains unclear. SAP is often characterized by recurrent episodes of inflammation and loss of tissue integrity in the intestine [15]. Gut microbiota is thought to be important for maintaining the balance of proinflammatory cytokines, which is closely associated with SAP progression. The knowledge for gut microbiota and its metabolites on intestinal barrier function in SAP will help us to understand the mechanism of gut failure in the pathogenesis of SAP [16]. Early dysbiosis of the gut microbiota is associated with the SAP risk, and the modulation of the gut microbiota is a potential approach in the prevention of SAP development [17].

Keap1-Nrf2-ARE ((Kelch-like ECH-associated protein 1) nuclear factor erythroid 2-related factor 2-antioxidant response element) are widely reported antioxidant signaling molecules [18, 19]. Pancreatitis is usually accompanied by the increase in the oxidative stress and inflammatory responses [20, 21]. Free radicals play an import role in the pathophysiology of SAP, and increased free radical activities and increased concentrations of lipid peroxides have been found in both SAP patients [22] and animal models [20, 23]. Xanthine oxidase (XO) [24] and nitric oxide synthase (NOS) [25] may be the individual contribution of possible sources of free radicals. Cellular oxidative stress contributes to pancreas injury and the improvement of antioxidant capacity by increasing the levels of the reduced glutathione (GSH) level, and catalase (CAT) activity will reduce the injury [26]. The enhancement of antioxidants superoxidase dismutase (SOD) and glutathione peroxidases (GPx) and the reduction of lipid oxidation product malondialdehyde (MDA) contribute to the recovery of SAP-induced intestinal barrier injury [27]. Therefore, the improvement of the expression of antioxidant signaling molecules will be a potential approach in the therapy of pancreatitis [28, 29]. Therefore, the aim of present work is to explore the protective effects of Saikosaponin A on SAP and delineate the underlying mechanisms of functional Saikosaponin A by investigating the composition of gut microbiota and related molecules.

## 2. Materials and Methods

### 2.1. Extracts of Saikosaponin A and Analysis of HPLC

Saikosaponin A standard (purity > 99%) was bought from Sigma (St. Louis, MO, USA) and dissolved in PBS buffer (20 mM, pH 7.4) with 0.1% BSA at room temperature. Sodium taurocholate was purchased from Sigma and dissolved in 0.9% NaCl to final concentration 1 mg/ml. All other reagents were analytical grade.

*Radix bupleuri* rhizomes were purchased from Shanghai State-owned Changning Pharmacy (Shanghai, China); Saikosaponin A was isolated and characterized according to a previous report with slight modification [30]. 1000-g rhizomes of *Radix bupleuri* were taken, crushed, sieved through a 20-30-mesh sieve, soaked with 100% ethanol for three days, filtered, combined with the soaking solution, and evaporated under reduced pressure on a rotary evaporator to recover ethanol. The sample was concentrated to obtain the total extracts of *Radix bupleuri*. The extracts were suspended in water and further extracted with petroleum ether, chloroform, and ethyl acetate to obtain a petroleum ether portion, a chloroform portion, an ethyl acetate portion, and a water-soluble portion, respectively. One thousand grams of macroporous resin H802 was packed into a column (10 × 60 cm). After pretreatment, ethyl acetate raffinate was added (50 g of the concentrated solution was dissolved in water, and insoluble matter was filtered off). After the sample loading, the column was washed with 3000 ml of water and then eluted with 50% ethanol, concentrated under reduced pressure on a rotary evaporator, and then dried in a vacuum oven. A total of 1500 g of 200~300 mesh silica gel was suspended in CHCl_3_ and equilibrated the column with CHCl_3_ in a column. The sample was dissolved in 200 ml of ethanol, mixed with 100 mesh silica gel, and dried at room temperature. After the column was equilibrated, the sample was loaded. CHCl3 : MeOH = 5 : 1 was used as the mobile phase to elute the sample successively. The eluted samples were collected (250 ml/bottle), numbered, and analyzed via high-pressure liquid chromatography (HPLC) (apparatus: HPLC-SCL-10 Avp; mobile phase: MeOH : H2O (4 : 6); detection wavelength: 265 nm; column size: Luna C18 250 × 4.6 mm; column temperature: 40°C; flow rate: 1 ml/min; and injection volume: 10 *μ*l). Thirty-nine grams of Saikosaponin was obtained. Finally, about 10 g of Saikosaponin A was finally purified on semipreparative HPLC (Beckman, Brea, CA, USA).

### 2.2. Establishment of the Model with SAP

**Figure 1:**
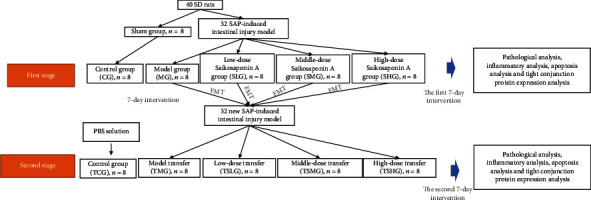
Study flow chart of the present experiment. There were 7 days for each stage, and the second stage was performed following the first stage.

Before the present study, all experimental processes were ensured to be consistent with the guidance for the care and use of laboratory animals from NIH and approved by the Animal Ethics Committee of The First Hospital of Jilin University (2017JLU0298). Eighty male Sprague-Dawley (SD) rats (8 weeks, 220–240 g) were purchased from the Animal Center of The First Hospital of Jilin University (Changchun, China). All rats were housed in separated cages (two rats in a cage) under either a 12 : 12 light : dark cycle (LD 12 : 12) and 22 ± 1°C with 65% humidity [2]. The rats had free access to standard food pellets and tap water ad libitum. SAP was established via the injection of 0.2 ml of 5% sodium taurocholate into the biliopancreatic duct according to the previous report [31]. SAP was induced by sodium taurocholate that resulted in acinar cell calcium overload, zymogen activation, cytokine activities, and cell death [32]. The SAP model was confirmed by the evaluated levels of blood lipase or amylase [33]. Fifty microliters blood was obtained from each rat tail vein before and after 3-day model establishment, and serum was prepared via centrifugation at 2000 × *g* for 10 min. Rat serum amylase and lipase were measured by using the rat pancreatic amylase (PAMY) ELISA Kit (Cat. No. MBS269618) and rat pancreatic lipase ELISA Kit (Cat. No. MBS453575) from MyBioSource (San Diego, CA, USA) according to the manufacturer's instructions. For the controls, the rats were injected with 0.9% NaCl solution. The animal model establishment was performed for twice as Figure 1 shows.

### 2.3. Animal Grouping

The dose range of Saikosaponin was referred to a report that the concentration of Saikosaponin A was from 6.25 mg/kg to 25.00 mg/kg [34], and a wider range of the dose (10, 20, and 40 mg/kg) was used in the present study. According to a previous report, pancreatic tissues were collected after 1-, 3-, and 5-day SAP model establishment. Most inflammatory cytokines had significant changes after 3- and 5-day SAP model establishment [35]. On the other hand, considering the effects of the Saikosaponin A on gut microbiota, a longer time (7 d) may be used [36]. There were two stages for the whole experiment as Figure 1 shows. After 12-hour sodium taurocholate injection, 24 SAP rats were administrated with different concentrations of Saikosaponin A (10, 20, and 40 mg/kg) in 0.2 ml 0.9% saline solution via the tail vein. Meanwhile, 0.2 ml 0.9% saline solution was injected in the control or animal group (*n* = 8 for each group) without Saikosaponin A. In the first stage, all rats were divided into CG (control group), MG (model group), SLG (low-dose Saikosaponin A), SMG (middle-dose Saikosaponin A), and SHG (high-dose Saikosaponin) groups according to different treatments (Figure 1). Rats were monitored daily for weight and disease activity index (DAI, including weight loss, presence of blood in feces, and stool consistency). The feces and urine samples were collected at 7 days. After 7 days, the rats were anesthetized with pentobarbital (40 mg/kg i.p.), blood samples were drawn, colon length was measured, and the animals were killed. Pancreas tissues were quickly removed and frozen at −80°C until use.

After 7-day Saikosaponin A intervention, fecal sample (1.0 g) was obtained from each rat in the MG, SLG, SMG, and SHG groups, suspended with 5 ml of sterile PBS buffer, and used to colonize the newly SAP rats. In the second stage, fecal microbiota transplantation (FMT) was transferred according to the previous study [37]. For the control group, the rats were treated with PBS buffer. Briefly, the fecal samples in the PBS solution (0.01 M, pH 7.4) were vortexed for 5 min, homogenized, and centrifuged for 10 min (1,000 g), and the pools were divided into equal volumes for 7 d. The model rats were colonized with pooled samples for another 7 d. These rats were divided into TCG, TMG, TSLG, TSMG, and TSHG groups according to the fecal microbiota were from MG, SLG, SMG, and SHG groups, respectively (Figure 1).

### 2.4. DAI

The DAI was evaluated according to the previous report, and the DAI score was presented as mean scores of body weight loss, fecal consistency, and fecal blood test scores [38].

### 2.5. Histological Evaluation

Colon tissues were washed in PBS buffer. The pancreas tissues were fixed by using 4% formalin, embedded in paraffin, cut in 4 m sections, and stained with hematoxylin and eosin (H&E) [39]. The inflammatory and injury of pancreas were observed in 5 fields per sections. The pathological grades were calculated according to Park's classification [40, 41] with slight modification: Grade 0, the lowest grade of injury with normal pancreas cell structure and without inflammation; Grade 1, the normal pancreas morphology was with a small number of mononuclear cell infiltration; Grade 2, the pancreas tissues were with middle number of mononuclear cell infiltration; Grade 3, the pancreas tissues were characterized with high-number of mononuclear cell infiltration; and Grade 4, pancreas structure was damaged with the significant number of mononuclear cell infiltration.

### 2.6. Serum Oxidative Assay

Blood sample was collected via abdominal aorta and centrifuged at 4000 × *g* for 10 min at 4°C. The serum was separated and stored at -80°C for subsequent biochemical testing. Sera MDA, CAT, SOD, and GPx were measured by using corresponding assay kits from Northwest Life Science Specialties (Vancouver, WA, USA), Cayman Chemical Company (MI, USA), Calbiochem (San Diego, CA, USA), and Nanjing Jiancheng Bio-Tek Co. (Nanjing, China) on an automatic blood chemical analyzer (CIBA Corning, OH, USA), respectively.

### 2.7. Serum Inflammatory Cytokines Analysis

The levels of TNF-*α* (SKU: BC-ER141303), IL-1*β* (SKU: BC-EH101933), IL-6 (SKU: BC-ER140741), and IL-10 (SKU: BC-ER140711) in serum were assessed by using ELISA kits following the manufacturer's scheme from Biocodon Technologies (Mission, KS, USA) and an automatic blood chemical analyzer (CIBA Corning, OH, USA). Serum C-reactive protein (CRP) was measured using the ELISA kit from DRG Instrument GmbH (Marburg, Germany) on a microplate reader from Thermo Scientific (Waltham, MA, USA). Serum procalcitonin (PCT) was measured with the ELISA kit from EIAab Science Co., Ltd. (Wuhan, China).

### 2.8. Reverse Transcription-Quantitative PCR (RT-qPCR)

RNA was isolated from 5 mg pancreas using TRIzol reagent (Shanghai Shenggong Co., Ltd., Shanghai, China). cDNA was made by using a reverse transcription kit (Shanghai Shenggong Co., Ltd., Shanghai, China). The following primers were used: Keap1 forward primer 5′-TTCGCCTACACGGCCTC-3′ and reverse primer 5′-GAAGTTGGCGATGCCGATG-3′; Nrf2, forward primer 5′-CCTCAACTATAGCGATGCTGAATCT-3′ and reverse primer 5′-AGGAGTTGGGCATGAGTGAGTAG-3′; ARE, forward primer 5′-CTGTCCTCAAATGAACCTGCCTCCTC-3′ and reverse primer 5′-GAGGAGGCAGGTTCCATTGAGGACAG-3′; and *β*-actin, forward primer 5′-AAGTCCCTCACCCTCCCAAAAG-3′ and reverse primer 5′-AAGCAATGCTGTCACCTTCCC-3′. RT-qPCR was performed on GeneAmp PCR System 9700 (Applied Biosystems, Foster City, CA, USA). The levels of target genes were detected and the relative mRNA levels were normalized to *β*-actin using the 2^-*ΔΔ*Ct^ method.

### 2.9. Western Blot

Pancreas tissues were used to measure the expression of Keap1, Nrf2, and ARE. Ten milligrams of pancreatic tissue was ground in liquid nitrogen, and total protein was extracted by using the Protein Isolation Kit (Invitrogen, CA, USA) according to the manufacture's instruction. Protein concentration was determined by using the BCA kit (Invitrogen, Carlsbad, CA, USA). HRP-conjugated goat anti-rabbit IgG H&L (ab6721) secondary antibodies were from Abcam (Abcam, San Francisco, CA, USA). The proteins were separated by SDS-PAGE and transferred to the PVDF membrane. The membrane was blocked for 1 hour at ambient room temperature in 10% nonfat milk and incubated with primary antibody (Anti-Keap1 antibody/Anti-Nrf2 antibody/Anti-ARE antibody from Abcam (1 : 1,000; Cambridge, MA, USA) overnight at 4°C. The membrane was rinsed 3 times with PBTB, incubated 2 hours at 37°C in secondary antibodies. Image was obtained on an infrared scanner (Odyssey, Lincoln, NE, USA). Relative protein levels were calculated by using internal reference *β*-actin.

### 2.10. Analysis of Gut Microbiota

Fecal pellets were collected and weighed, homogenized with 1 ml of sterile PBS. Bacterial DNA was extracted from the samples by using the QIAamp Fast DNA Stool Mini Kit (catalog number: 51604, QIAGEN, CA, USA). The isolated DNA was amplified using primers for the target gene 16S rRNA (V3-4 regions: forward 5′-CCTACGGGNGGCWGCAG-3′ and reverse 5′-GACTACHVGGGTATCTAATCC-3′) according to the previous report [42]. The gut microbiota of rats was analyzed by using 16S rRNA sequencing of bacterial genomes.

### 2.11. Statistical Analysis

All data were presented as the mean ± standard deviation (S.D.). The variables were analyzed by using unpaired two tailed Student's *t*-test. A normal distribution of variance was confirmed by the Kolmogorov and Smirnov test [43]. Homogeneity of variance was confirmed by using Bartlett's test [44]. *p* values were corrected for multiple comparisons by using the Bonferroni adjustment [45]. The statistical data were analyzed by one-way ANOVA followed by Tukey's post hoc test. The statistical difference was considered if *p* < 0.5.

## 3. Results

### 3.1. HPLC Analysis of Saikosaponin A

**Figure 2:**
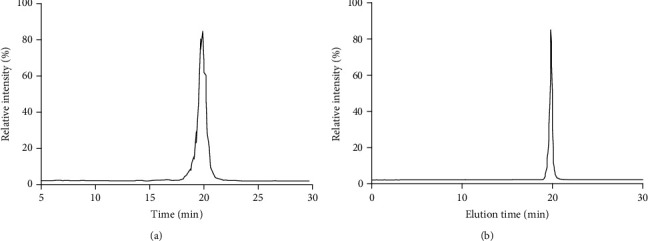
High-performance liquid chromatography (HPLC) analysis for the extracts of *Radix bupleuri*. (a) The standard of Saikosaponin A. (b) Purified Saikosaponin A.

Comparing with the standard of Saikosaponin A (Figure 2(a)), HPLC analysis showed that the main extracts of *Radix bupleuri* was Saikosaponin A (Figure 2(b)) and the eluting time was 19.9 min. *Radix bupleuri* may exert its function via its main component Saikosaponin A.

### 3.2. Saikosaponin A Intervention Reduced SAP Symptoms

**Figure 3:**
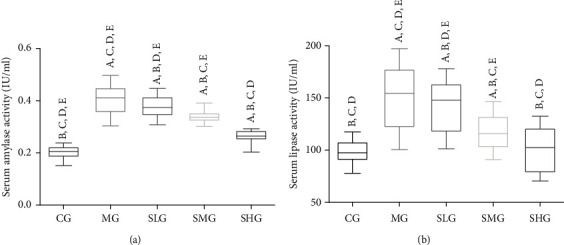
The effects of Saikosaponin A on serum lipase and amylase among different groups. Data were presented as means ± S.D. (standard deviation) and *n* = 8 for each group. ^a^*p* < 0.05 vs. the CG group, ^b^*p* < 0.05 vs. the MG group, ^c^*p* < 0.05 vs. the SLG group, ^d^*p* < 0.05 vs. the SMG group, and ^e^*p* < 0.05 vs. the SHG group.

The present results showed that the levels of serum amylase (Figure 3(a)) and serum lipase (Figure 3(b)) significantly increased in the MG group when compared with the CG group (*p* < 0.05). The results suggested that SAP was established with the significantly increased levels of serum amylase and lipase. On the other hand, Saikosaponin A treatment reduced the level of serum amylase (Figure 3(a)) and serum lipase (Figure 3(b)) in a dose-dependent way (*p* < 0.05). The results suggest that Saikosaponin A intervention reduces serum levels of amylase and lipase in the SAP model.

**Figure 4:**
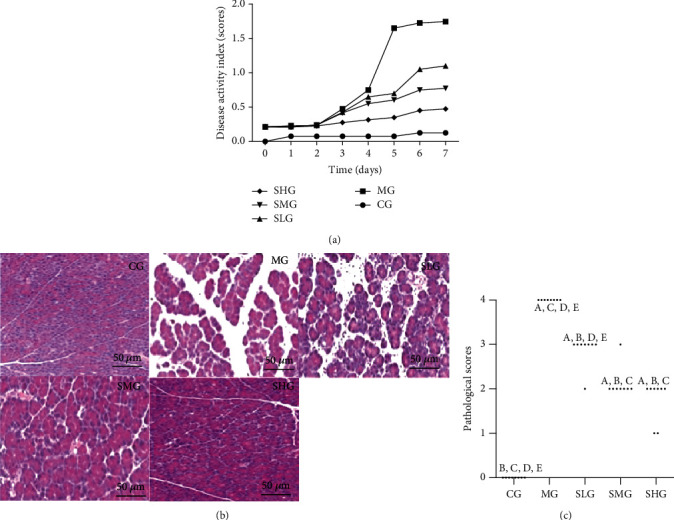
Saikosaponin A ameliorated pathological character of SAP in rats. (a) Disease activity index. (b) Hematoxylin and eosin (H&E) staining of pancreas in each group. (c) Histopathological scores. Data were presented as means ± S.D. (standard deviation) and *n* = 8 for each group. ^a^*p* < 0.05 vs. the CG group, ^b^*p* < 0.05 vs. the MG group, ^c^*p* < 0.05 vs. the SLG group, ^d^*p* < 0.05 vs. the SMG group, and ^e^*p* < 0.05 vs. the SHG group.

To explore the effects of Saikosaponin A on pancreas damage, an SAP model was established in rats. The DAI was 0 in the CG group, and the scores were highest in the MG group. Saikosaponin A intervention reduced the DAI value in a dose-dependent way (Figure 4(a), *p* < 0.05). Pathological change was assessed by using the H&E stain. There was no pathological character in the CG group with Grade 0. In contrast, the rats had a significant pathological character in the MG group with grade 4, including acinar cell edema, widened intercellular spaces, hemorrhage, necrosis, inflammatory cell infiltration, and cell destruction (Figures 4(b) and 3(c), *p* < 0.05). The pathological characters were significantly reduced in Saikosaponin A-treated group in a dose-dependent way (Figures 4(b) and 3(c), *p* < 0.05). These results suggest that Saikosaponin A intervention ameliorates SAP lesions.

### 3.3. Saikosaponin A Had Antioxidant and Anti-Inflammatory Effects on the Rats with SAP

**Figure 5:**
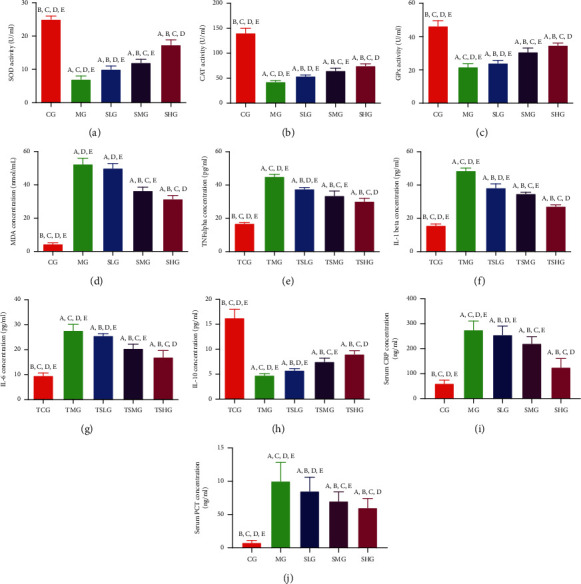
The effects of administration of Saikosaponin A on serum level of oxidative stress, inflammatory responses, and apoptosis status in the rats with severe acute pancreatitis (SAP). (a) Superoxide dismutase (SOD). (b) Catalase (CAT). (c) Oxidized glutathione (GPx). (d) Malondialdehyde (MDA). (e) Tumor necrosis factor- (TNF-) *α*. (f) Interleukin- (IL-) 1*β*. (g) IL-6. (h) IL-10. (i) C-reactive protein (CRP). (j) Procalcitonin (PCT). Data were presented as means ± S.D. (standard deviation) and *n* = 8 for each group. ^a^*p* < 0.05 vs. the CG group, ^b^*p* < 0.05 vs. the MG group, ^c^*p* < 0.05 vs. the SLG group, ^d^*p* < 0.05 vs. the SMG group, and ^e^*p* < 0.05 vs. the SHG group.

Antioxidant analysis showed that serum levels of SOD (Figure 5(a)), CAT (Figure 5(b)), and GPx (Figure 5(c)) were highest in the CG group and significantly reduced in the MG group while the MDA level was lowest in the CG group and highest in the MG group (Figure 5(d), *p* < 0.05). Saikosaponin A intervention increased the serum levels of SOD (Figure 5(a)), CAT (Figure 5(b)), and GPx (Figure 5(c)) and reduced the MDA level (Figure 5(d), *p* < 0.05). The administration of Saikosaponin A suppressed oxidative stress in the rats with SAP.

Anti-inflammatory analysis showed that serum levels of TNF-*α* (Figure 5(e)), IL-1*β* (Figure 5(f)), and IL-6 (Figure 5(g)) were lowest in the CG group and significantly increased in the MG group while the IL-10 level was highest in the CG group and lowest in the MG group (Figure 5(h), *p* < 0.05). Saikosaponin A intervention reduced the serum levels of TNF-*α* (Figure 5(e)), IL-1*β* (Figure 5(f)), and IL-6 (Figure 5(g)) and increased the IL-10 level (Figure 5(h), *p* < 0.05). The levels of serum CRP (Figure 5(i)) and serum PCT (Figure 5(j)) significantly increased in the MG group when compared with the CG group (*p* < 0.05). The results suggested that SAP was established with the significantly increased levels of serum CRP and PCT. On the other hand, Saikosaponin A treatment reduced the level of serum CRP (Figure 5(i)) and serum PCT (Figure 5(j)) in a dose-dependent way (*p* < 0.05). The results suggest that Saikosaponin A intervention reduces serum levels of inflammatory indicators in the SAP model. Saikosaponin A increased anti-inflammatory properties in the rats with SAP.

### 3.4. Saikosaponin A Increased the Relative mRNA Levels of Antioxidant Signaling Molecules

**Figure 6:**
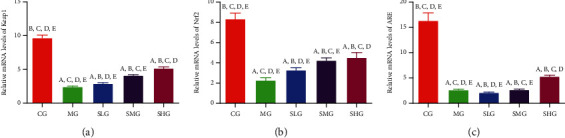
The real-time reverse transcription polymerase chain reaction (RT-PCR) analysis of the effects of Saikosaponin A on the relative mRNA levels of antioxidant signaling proteins. (a) Kelch-like ECH-associated protein 1 (Keap1). (b) Nuclear factor erythroid 2-related factor 2 (NRF2). (c) Antioxidant response element (ARE). Data were presented as mean values ± S.D. (standard deviation) and *n* = 8 for each group. ^a^*p* < 0.05 vs. the CG group, ^b^*p* < 0.05 vs. the MG group, ^c^*p* < 0.05 vs. the SLG group, ^d^*p* < 0.05 vs. the SMG group, and ^e^*p* < 0.05 vs. the SHG group.

Keap1-Nrf2-ARE are important antioxidant signaling molecules to maintain antioxidant properties and pancreas integrity [46]. Thus, we explored the protective function by investigating the effects of Saikosaponin A on the relative mRNA levels of the antioxidant signaling. The levels of Keap1 (Figure 6(a)), Nrf2 (Figure 6(b)), and ARE (Figure 6(c)) were obviously decreased in the MG group when compared with those in the CG group (*p* < 0.05). However, the administration of Saikosaponin A increased the level of Keap1 (Figure 6(a)), Nrf2 (Figure 6(b)), and ARE (Figure 6(c)). The experiment result demonstrated that Saikosaponin A ameliorated the SAP by increasing the relative mRNA levels of antioxidant signaling molecules.

### 3.5. Saikosaponin A Increased the Protein Levels of Antioxidant Signaling Molecules

**Figure 7:**
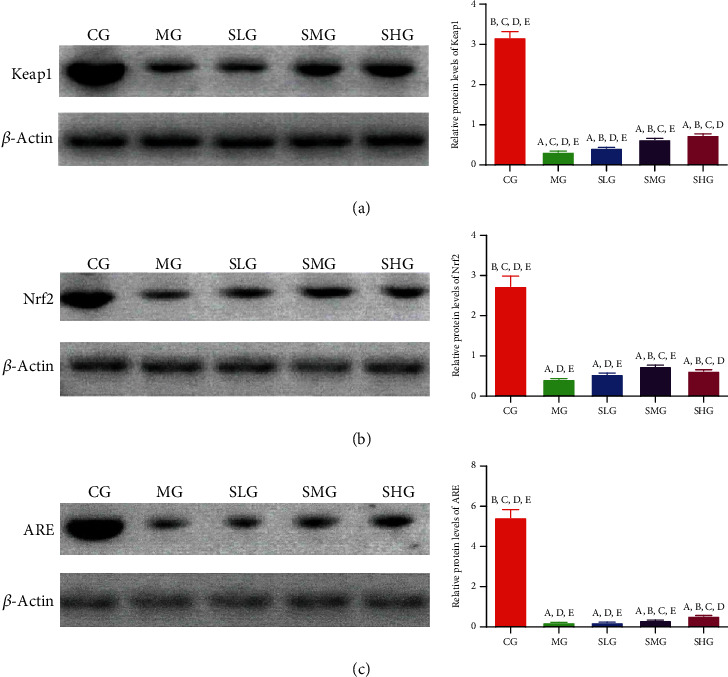
Western blot analysis of the effects of Saikosaponin A on the expression of antioxidant signaling proteins. (a) Kelch-like ECH-associated protein 1 (Keap1). (b) Nuclear factor erythroid 2-related factor 2 (NRF2). (c) Antioxidant response element (ARE). Data were presented as mean values ± S.D. (standard deviation) and *n* = 8 for each group. ^a^*p* < 0.05 vs. the CG group, ^b^*p* < 0.05 vs. the MG group, ^c^*p* < 0.05 vs. the SLG group, ^d^*p* < 0.05 vs. the SMG group, and ^e^*p* < 0.05 vs. the SHG group.

We further explored the protective function by investigating the effects of Saikosaponin A on the protein levels of the antioxidant signaling molecules. The expression levels of Keap1 (Figure 7(a)), Nrf2 (Figure 7(b)), and ARE (Figure 7(c)) obviously decreased in the MG group when compared with those in the CG group (*p* < 0.05). However, the administration of Saikosaponin A increased the expression levels of Keap1 (Figure 7(a)), Nrf2 (Figure 7(b)), and ARE (Figure 7(c)). The experiment result demonstrated that Saikosaponin A ameliorated the SAP status by increasing the expression of antioxidant signaling proteins.

### 3.6. Saikosaponin A Intervention Improved Gut Microbiota Composition

**Figure 8:**
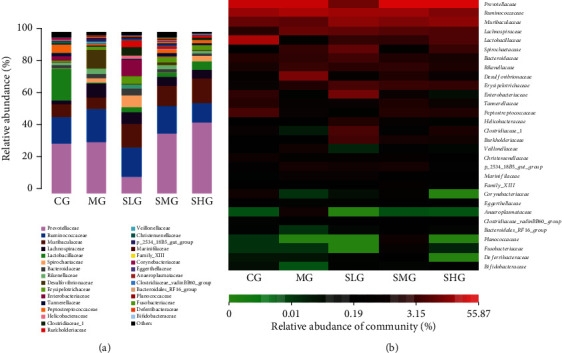
The composition of gut microbiota among different groups. (a) The proportion of gut microbiota. (b) Heatmap analysis of gut microbiota changes from different treatments.

Dysbiosis of gut microbiota is closely associated with pancreas dysfunction and damage. To investigate the impact of Saikosaponin A on gut microbiota composition, 16S rRNA gene sequencing was conducted by using the different fecal specimens from all groups. The relative abundance of Lactobacillus species was highest in the CG, and lowest in the MG group (Figure 8(a)). Saikosaponin A treatment increased the relative abundance of Lactobacillus species in a dose-dependent way (Figure 8(a)). The abundance of Prevotella species was similar between the CG and MG groups (Figure 8(a)). Saikosaponin A treatment increased the relative abundance of Prevotella species in a dose-dependent way (Figure 8(a)). Heatmap analysis showed the similar results as those in the barplots (Figure 8(b)).

### 3.7. FMT of Saikosaponin A-Treated Rats Ameliorated SAP Lesions

**Figure 9:**
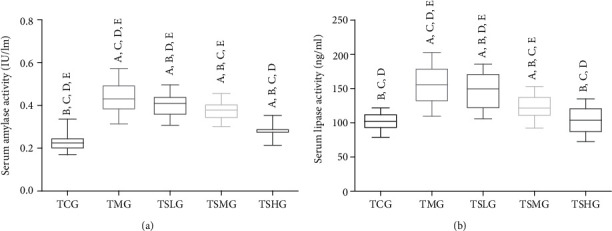
The effects of fecal microbiota transplantation (FMT) of Saikosaponin A-treated rats on serum lipase and amylase among different groups. Data were presented as means ± S.D. (standard deviation) and *n* = 8 for each group. ^a^*p* < 0.05 vs. the TCG group, ^b^*p* < 0.05 vs. the TMG group, ^c^*p* < 0.05 vs. the TSLG group, ^d^*p* < 0.05 vs. the TSMG group, and ^e^*p* < 0.05 vs. the TSHG group.

**Figure 10:**
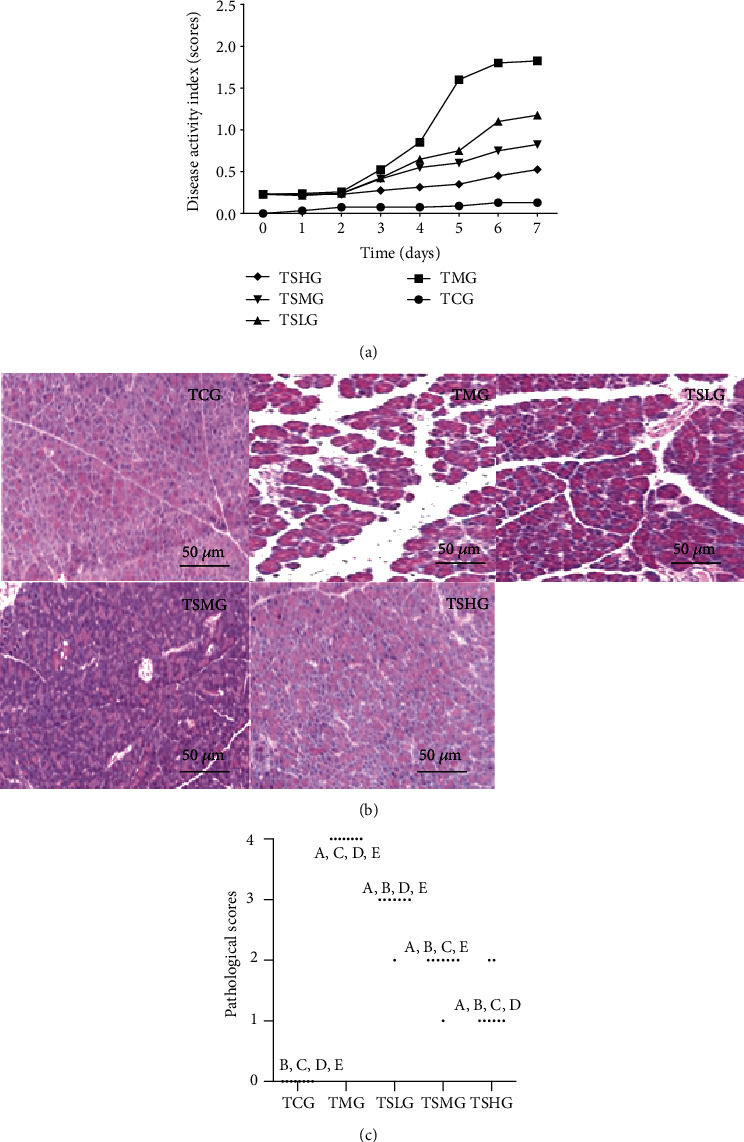
Fecal microbiota transplantation (FMT) of Saikosaponin A-treated rats ameliorated pathological character of severe acute pancreatitis (SAP) in rats. (a) Disease activity index. (b) Hematoxylin and eosin (H&E) staining of pancreas in each group. (c) Histopathological scores. Data were presented as means ± S.D. (standard deviation) and *n* = 8 for each group. ^a^*p* < 0.05 vs. the TCG group, ^b^*p* < 0.05 vs. the TMG group, ^c^*p* < 0.05 vs. the TSLG group, ^d^*p* < 0.05 vs. the TSMG group, and ^e^*p* < 0.05 vs. the TSHG group.

To explore whether the gut microbiota of Saikosaponin A-treated animals improve the SAP rats, the gut microbiota of Saikosaponin A-treated SAP rats (the model was established within the first 7 days) was transferred to another SAP rats (the model was established within the second 7 days). All the previous parameters were repeated analyzed. The levels of serum amylase (Figure 9(a)) and serum lipase (Figure 9(b)) significantly increased in the TMG group when compared with the TCG group (*p* < 0.05). The results suggested that FMT of the SAP model rats significantly increased the levels of serum amylase and lipase. On the other hand, FMT of Saikosaponin A-treated rats reduced the level of serum amylase (Figure 9(a)) and serum lipase (Figure 9(b), *p* < 0.05). The results suggest that FMT of Saikosaponin A-treated rats reduces serum levels of amylase and lipase in the SAP model. FMT of model rat increased disease activity index scores (Figure 10(a)) and pathological scores (Figures 10(b) and 10(c)). In contrast, the FMT of Saikosaponin A-treated rats reduced disease activity index scores (Figure 10(a)) and pathological scores (Figures 10(b) and 10(c)). These results suggest that Saikosaponin A ameliorates SAP lesions by improving gut microbiota.

### 3.8. FMT of Saikosaponin A-Treated Rats Had Antioxidant and Anti-Inflammatory Effects on the Rats with SAP

**Figure 11:**
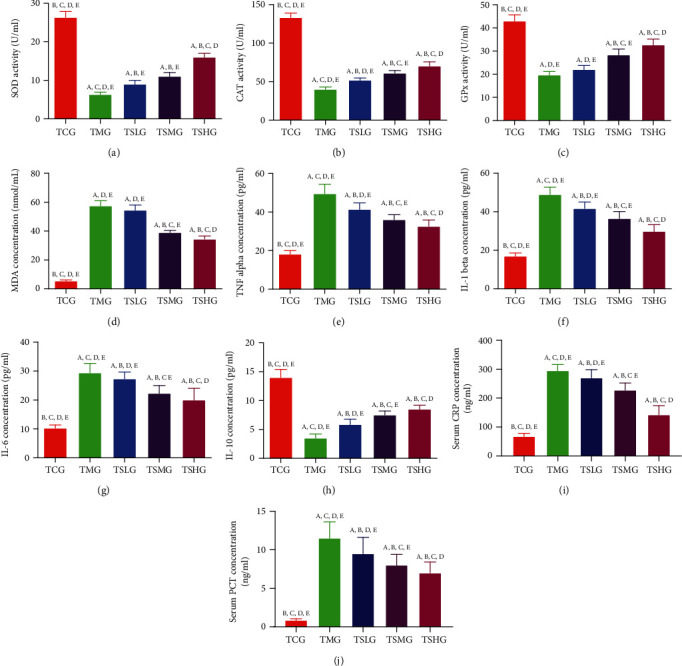
The effects of Saikosaponin A-treated rats on serum level of oxidative stress, inflammatory responses, and apoptosis status in the rats with severe acute pancreatitis (SAP). (a) Superoxide dismutase (SOD). (b) Catalase (CAT). (c) Oxidized glutathione (GPx). (d) Malondialdehyde (MDA). (e) Tumor necrosis factor- (TNF-) *α*. (f) Interleukin- (IL-) 1*β*. (g) IL-6. (h) IL-10. (i) C-reactive protein (CRP). (j) Procalcitonin (PCT). Data were presented as means ± S.D. (standard deviation) and *n* = 8 for each group. ^a^*p* < 0.05 vs. the TCG group, ^b^*p* < 0.05 vs. the TMG group, ^c^*p* < 0.05 vs. the TSLG group, ^d^*p* < 0.05 vs. the TSMG group, and ^e^*p* < 0.05 vs. the TSHG group.

Antioxidant analysis showed that serum levels of SOD (Figure 11(a)), CAT (Figure 11(b)), and GPx (Figure 11(c)) were highest in the TCG group and significantly reduced in the TMG group while the MDA level was lowest in the TCG group and highest in the TMG group (Figure 11(d), *p* < 0.05). FMT of Saikosaponin A-treated rats increased the serum levels of SOD (Figure 11(a)), CAT (Figure 11(b)), and GPx (Figure 11(c)) and reduced the MDA level (Figure 11(d), *p* < 0.05). FMT of Saikosaponin A-treated rats suppressed oxidative stress in the rats with SAP.

Anti-inflammatory analysis showed that serum levels of TNF-*α* (Figure 11(e)), IL-1*β* (Figure 11(f)), and IL-6 (Figure 11(g)) were lowest in the TCG group and significantly increased in the TMG group while the IL-10 level was highest in the TCG group and lowest in the TMG group (Figure 11(h), *p* < 0.05). FMT of Saikosaponin A-treated rats reduced the serum levels of serum levels of TNF-*α* (Figure 11(e)), IL-1*β* (Figure 11(f)), and IL-6 (Figure 11(g)) and increased the IL-10 level (Figure 11(h), *p* < 0.05). The levels of serum CRP (Figure 11(i)) and serum PCT (Figure 11(j)) significantly increased in the TMG group when compared with the TCG group (*p* < 0.05). The results suggested that FMT of SAP model rats significantly increased the levels of serum CRP and PCT. On the other hand, FMT of Saikosaponin A-treated rats reduced the level of serum CRP (Figure 11(i)) and serum PCT (Figure 11(j), *p* < 0.05). FMT of Saikosaponin A-treated rats increased anti-inflammatory properties in the rats with SAP.

### 3.9. FMT of Saikosaponin A-Treated Rats Increased the Relative mRNA Levels of Antioxidant Signaling Protein

**Figure 12:**
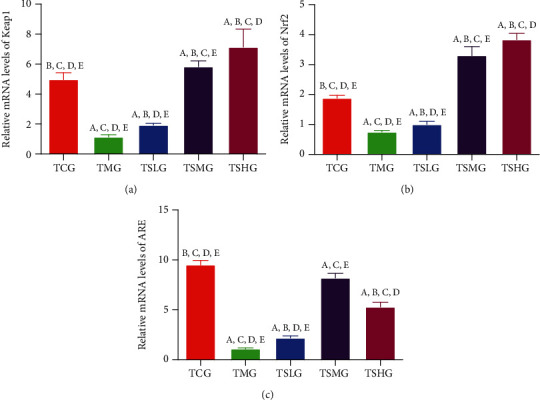
The real-time reverse transcription polymerase chain reaction (RT-PCR) analysis of the effects of fecal microbiota transplantation (FMT) of Saikosaponin A on relative mRNA levels of antioxidant signaling proteins. (a) Kelch-like ECH-associated protein 1 (Keap1). (b) Nuclear factor erythroid 2-related factor 2 (NRF2). (c) Antioxidant response element (ARE). Data were presented as means ± S.D. (standard deviation) and *n* = 8 for each group. ^a^*p* < 0.05 vs. the TCG group, ^b^*p* < 0.05 vs. the TMG group, ^c^*p* < 0.05 vs. the TSLG group, ^d^*p* < 0.05 vs. the TSMG group, and ^e^*p* < 0.05 vs. the TSHG group.

The relative mRNA levels of Keap1 (Figure 12(a)), Nrf2 (Figure 12(b)), and ARE (Figure 12(c)) was obviously decreased in the TMG group when compared with those in the TCG group (*p* < 0.05). However, FMT of Saikosaponin A-treated rats increased the relative mRNA levels of Keap1 (Figure 12(a)), Nrf2 (Figure 12(b)), and ARE (Figure 12(c)). The experiment result demonstrated that FMT of Saikosaponin A-treated rats ameliorated the SAP by increasing the relative mRNA levels of antioxidant signaling molecules.

### 3.10. FMT of Saikosaponin A-Treated Rats Increased the Expression of Antioxidant Signaling Protein

**Figure 13:**
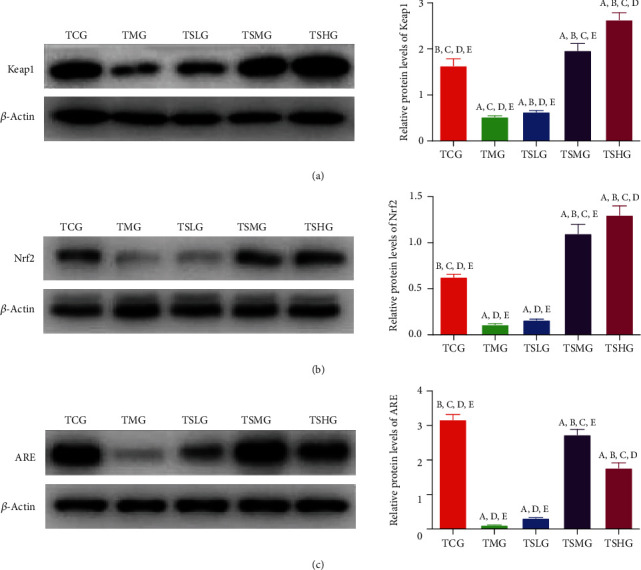
Western blot analysis of the effects of FMT of Saikosaponin A on the expression of antioxidant signaling proteins. (a) Kelch-like ECH-associated protein 1 (Keap1). (b) Nuclear factor erythroid 2-related factor 2 (NRF2). (c) Antioxidant response element (ARE). Data were presented as means ± S.D. (standard deviation) and *n* = 8 for each group. ^a^*p* < 0.05 vs. the TCG group, ^b^*p* < 0.05 vs. the TMG group, ^c^*p* < 0.05 vs. the TSLG group, ^d^*p* < 0.05 vs. the TSMG group, and ^e^*p* < 0.05 vs. the TSHG group.

Western blot analysis showed that the expression levels of Keap1 (Figure 13(a)), Nrf2 (Figure 13(b)), and ARE (Figure 13(c)) obviously decreased in the TMG group when compared with the TCG group (*p* < 0.05). However, FMT of Saikosaponin A-treated rats increased the expression levels of Keap1 (Figure 13(a)), Nrf2 (Figure 13(b)), and ARE (Figure 13(c)). The experiment result demonstrated that FMT of Saikosaponin A-treated rats ameliorated the SAP by increasing the expression of antioxidant signaling protein.

### 3.11. FMT of Saikosaponin A-Treated Rats Improved Gut Microbiota Composition

**Figure 14:**
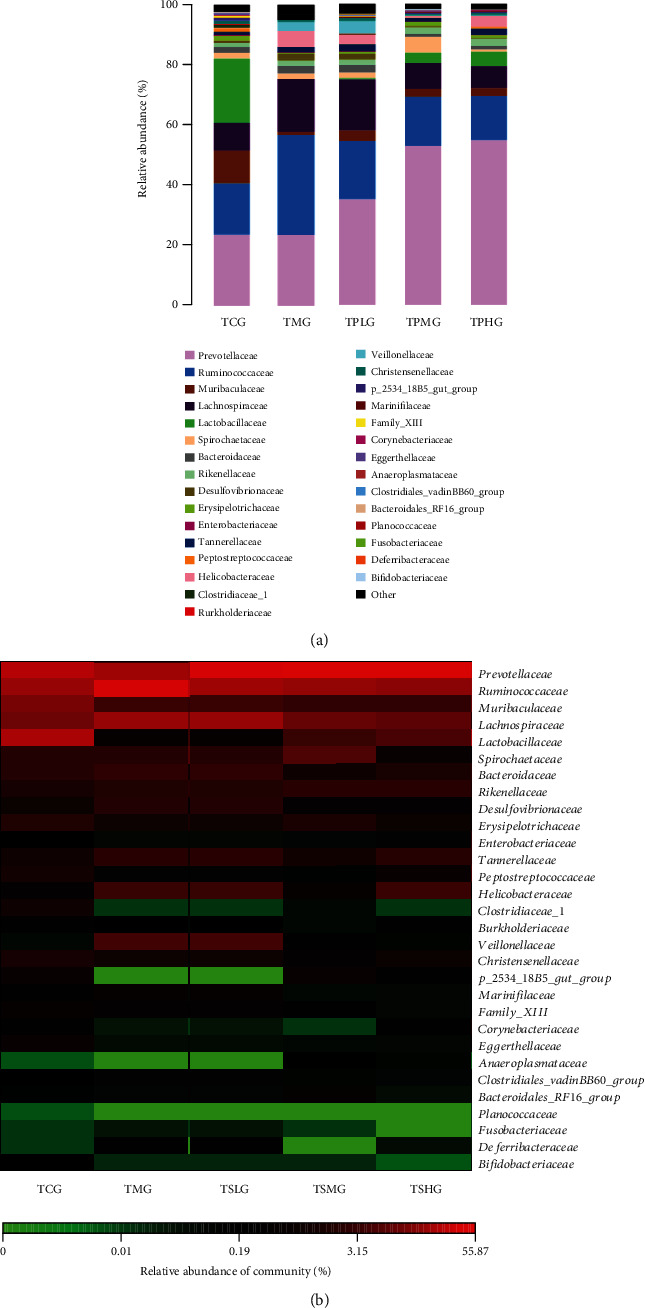
The effects of FMT on composition of gut microbiota among different groups. (a) The proportion of gut microbiota. (b) Heatmap analysis of gut microbiota changes from different FMT.

The abundance of Lactobacillus species was highest in the TCG, and lowest in the TMG group (Figure 14(a)). FMT of Saikosaponin A-treated rats increased the relative abundance of Lactobacillus species (Figure 14(a)). The abundance of Prevotella species was similar between the TCG and TMG groups (Figure 14(a)). FMT of Saikosaponin A-treated rats increased the relative abundance of Prevotella species in a dose-dependent way (Figure 14(a)). Heatmap analysis showed the similar results as the barplots (Figure 14(b)). Therefore, FMT of Saikosaponin A-treated rats improved the gut microbiota by increasing the proportion of Lactobacillus and Prevotella species (Figure 14). The results indicated that FMT of Saikosaponin A-treated rats improved gut microbiota in the SAP model.

## 4. Discussion

SAP lesions are linked with sepsis, infected pancreatic necrosis, and multiorgan failure [47]. SAP is a relapsing complication of digestive system and can lead to chronic inflammatory disease [48]. Many drugs are used for the treatment of SAP but most of them have unwanted adverse effects [49]. The present work showed that Saikosaponin A exerted protective effects against SAP risk by reducing pathological scores (Figure 4) and increasing antioxidant and anti-inflammation (Figure 5) properties. We next examined the effects of Saikosaponin A on the expression of antioxidant signaling molecules. Saikosaponin A administration significantly improved the expression of Keap1, Nrf2, and ARE (Figures 7 and 8). Furthermore, Saikosaponin A intervention also increased anti-inflammatory capacity by reducing the levels of IL-6, IL-1*β*, and TNF-*α* and increased the level of IL-10 (Figure 5). The results were consistent with the previous reports that Saikosaponin A treatment reduced the IL-6, IL-1*β*, and TNF-*α* levels [50] and increased IL-10 level [50, 51]. Saikosaponin A treatment also reduced the levels of main inflammatory factors of SAP, CRP, and PCT (Figure 5), but the related report was not found yet. These results suggest that Saikosaponin A is a potential drug in the prevention of SAP progression.

Saikosaponin A treatment improved antioxidant capacity by increasing the serum levels of SOD, CAT, and GPx and reducing the MDA level. Free radicals and reactive oxygen species (ROS) and reactive nitrogen species (RNS) contribute to many pancreatitis processes by inducing oxidative stress and oxidative damage [29, 52, 53]. The increase in the ROS level induces DNA damage and potential cytotoxicity [54]. SOD shows its antioxidant role by directly scavenging excess intracellular free radicals and reducing MDA level and enhancing total antioxidant capacity (T-AOC) [55]. Decreased antioxidant SOD activity will induce the increase in the levels of oxidative stress biomarkers (MDA) in the SAP model [56]. GPx is a selenium-dependent enzyme that prevents intracellular hydrogen peroxide and lipid peroxides [57]. CAT widely exists in mammalian cells and shows the protection against from ROS, which is produced through the decomposition of H_2_O_2_ [58]. Serum activity of GPx and SOD is closely associated with the removal of ROS [59]. SOD, CAT, and GPx are indispensable in the defense against oxidative species into bloodstream, especially super oxide anion radical (O_2_·-), which is continuously produced in human body metabolism via the mitochondrial energy production pathway [60].

The ROS superoxide anion radical is mainly generated by NADPH oxidase (NOX) in the SAP model and usually converted into H_2_O_2_ with the participation of SOD, as well as NO generated by inducible nitric oxide synthase. The latter ROS generated from XO plays a crucial role in SAP injury [29]. The previous reports showed that Saikosaponin A reduced the expression of NOX [61]. The extracts with Saikosaponin A inhibited the superoxide anion formation by XO [62].

Nrf2-ARE pathway also attenuates oxidative stress-induced DNA damage in pancreatic beta cells [63]. Keap1-Nrf2-ARE are important antioxidant signaling and exerts protective function against SAP. The expression of Keap1-Nrf2-ARE was significantly downregulated in SAP rats. The change was inhibited via Saikosaponin A administration. Many antioxidant enzyme systems are expressed by activating the Keap1-Nrf2-ARE signaling pathway [64]. These results indicate that Saikosaponin A may exert an important protective effect on pancreas integrity by affecting the expression of Keap1-Nrf2-ARE antioxidant signaling.

On the other hand, the treatment of Saikosaponin A treatment improved gut microbiota composition (Figure 8). Saikosaponin A may show its function via the improvement of gut microbiota composition, and the results of FMT further confirmed such a proposal. To explore the effects of FMT on pancreatitis, histology analysis was also performed. FMT of Saikosaponin A-treated rats increased colon length and reduced DAI and pathological scores. FMT of Saikosaponin A-treated rats also increased antioxidant and anti-inflammation properties (Figure 11). Meanwhile, the FMT of Saikosaponin A-treated rats increased the expression of antioxidant signaling molecules of Keap1-Nrf2-ARE (Figure 13).

**Figure 15:**
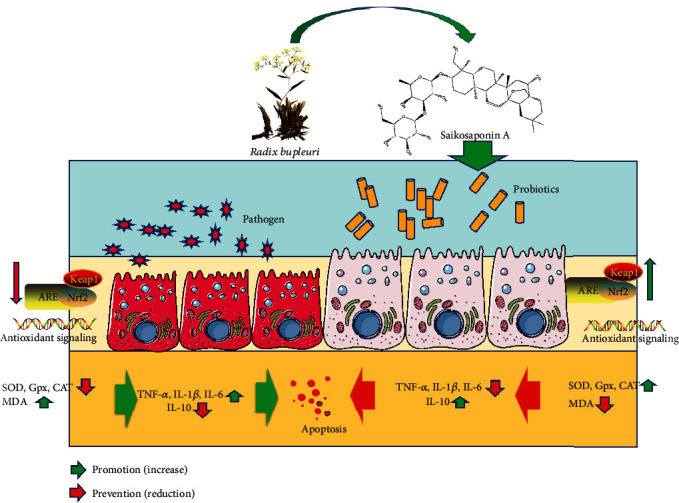
Saikosaponin A from *Radix bupleuri* prevented severe acute pancreatitis. Saikosaponin A intervention improved gut microbiota composition, which reduces inflammation responses and improves antioxidant properties via Kelch-like ECH-associated protein 1-nuclear factor erythroid 2-related factor 2-antioxidant response element (Keap1-Nrf2-ARE) signaling.

Additionally, Saikosaponin A intervention improved gut microbiota and showed significant increases in the probiotics, including Lactobacillus and Prevotella species (Figure 8). Similarly, FMT of Saikosaponin A-treated rats also improved gut microbiota. FMT-treated samples showed significant increases in the probiotics, including Lactobacillus and Prevotella species (Figure 14). Lactobacillus species suppressed and repair *E. coli*-impaired SAP by increasing the expression and distribution of antioxidant proteins and can be served as an essential food additive to solve health complications. Lactobacillus as a new generation of probiotics plays an important role in maintaining intestinal epithelial homeostasis and exerting health-promoting function [65]. Prevotella species may exert their function by fermenting carbohydrate and releasing short-chain fatty acid (SCFA) to protect pancreas integrity [66]. Notably, *Prevotella copri* is potential harmful bacterium which can be inhibited by lactobacillus bacteria [67]. Surprisingly, the abundance of Lactobacillus and Prevotella species was increased after supplementation with Saikosaponin A or gavage with FMT. These results suggest that Saikosaponin A-induced gut microbiota reduces SAP by reducing inflammatory responses and improving antioxidant signaling through gut microbiota (Figure 15).

There were some limitations in the present study. The redox homeostasis was only analyzed in the blood sample although it is more important to measure the redox homeostasis in pancreatic tissues for better understanding the effects of Saikosaponin A on the SAP risk. The serum concentrations of ROS and RNS were not measured although ROS and RNS levels are closely associated with oxidative stress. Albumin is a very abundant and important circulating antioxidant with ligand binding and strong DPPH (1,1-diphenyl-2-picrylhydrazyl) radical scavenging activity properties [68, 69]. However, serum albumin was not measured in the present experiment. Furthermore, only the activities of selected redox biomarkers were evaluated without considering other critical redox protein, DNA oxidation products, ROS production rate, etc. The effects of FMT on healthy rats were not evaluated. Finally, the rat models could not adequately imitate the clinical scenarios of human SAP. The effects of Saikosaponin A treatment on microbiota-depleted rats were not explored in the present work, and thus, the function of Saikosaponin A may be affected by the preexisting gut microbiota. Saikosaponin A intervention may induce the production of an antimicrobial peptide (AMP), which can change the composition of gut microbiota by reducing the bacterial translocation. The decrease in the AMP levels will result in the dysbiosis of gut microbiota in the AP model [70]. The FMT may contain such AMP and affect the distribution of gut microbiota. Therefore, the effect of FMT in normal animals should be studied if AMP can be identified and used to treat AP. Further work is needed to address these important issues.

In conclusion, this study indicates that administration of Saikosaponin A intervention attenuates SAP lesions. Saikosaponin A treatment not only increased antioxidant and anti-inflammatory properties but also improved SAP lesions in the rat model. These findings indicate that Saikosaponin A-induced gut microbiota changes may have a potential protective effect on SAP and are useful in the prevention of the inflammatory disease as SAP.

## Data Availability

All data are available from the corresponding author (Yanfang Jiang, Email: jiangyfjl@126.com) upon reasonable request.
